# Understanding Exercise Facilitators, Preferences, and Goals in Females With Type 2 Diabetes: A Mixed-Methods Analysis

**DOI:** 10.1177/26350106261475949

**Published:** 2026-08-31

**Authors:** Sian A. O’Gorman, Bridgette M. Desjardins, Michelle A. Keske, Kimberley L. Way

**Affiliations:** 1 Faculty of Health, School of Exercise and Nutrition Sciences, Deakin University, Burwood, Australia; 2 Institute for Physical Activity and Nutrition, Deakin University, Geelong, Australia; 3 Baker Heart and Diabetes Institute, Melbourne, Victoria, Australia; 4 University of Ottawa Heart Institute, Ottawa, Ontario, Canada

**Keywords:** type 2 diabetes, women, physical activity, exercise self-management, exercise preferences

## Abstract

**Purpose:**

The purpose of this study was to examine facilitators, preferences, and goals influencing exercise self-management among Australian females with type 2 diabetes (T2D) to inform more person-centered exercise counselling and support.

**Methods:**

This was a mixed-methods analysis of females with self-reported T2D (≥18 years) who completed an anonymous online survey regarding exercise self-management. The survey included quantitative items assessing demographics, physical activity levels, exercise facilitators, and exercise preferences and free-text responses that were thematically analyzed to explore exercise goals. Responses were also compared by age group (younger: 18-54 years; older: ≥55 years) to explore potential differences across adulthood.

**Results:**

Participants (n = 119) were aged 22 to 87 years (mean: 59.5 ± 13.3 years), with a median T2D duration of 4.1 years. Most (57%) reported being physically active ≥150 min/wk. Participants were primarily motivated by the perceived health benefits of exercise, particularly weight and glycemic management; however, qualitative insights revealed that these were often framed as strategies to reduce the need for medication. Exercise facilitators, preferences, and goals were largely consistent across age groups.

**Conclusion:**

These findings highlight opportunities to better align exercise counselling and support with the priorities of females with T2D. Educating females about the broader benefits of exercise beyond weight and glycemia and framing it as a strategy to reduce medication burden may enhance its perceived relevance and uptake. The consistency of findings across age groups suggests that strategies to support exercise self-management may be broadly applicable across adulthood.

## Introduction

Exercise is a cornerstone of type 2 diabetes (T2D) self-management due to its benefits on cardiometabolic risk factors, including insulin resistance, hyperglycemia, blood pressure, adiposity, lipid profile, and cardiorespiratory fitness.^1^ Given that cardiovascular disease (CVD) remains the leading cause of morbidity and mortality in individuals with T2D,^2^ optimizing exercise engagement is paramount to reducing long-term risk. Females with T2D experience a disproportionately higher relative risk of CVD and cardiovascular mortality compared to males with T2D,^3^ yet they are the least likely to meet physical activity (PA) guidelines.^4^ This highlights a critical gap between the recognized importance of exercise self-management and its real-world uptake in this high-risk population.

Behavioral research in T2D has largely focused on identifying exercise barriers, including those particularly salient for females, such as low motivation, low self-efficacy, inadequate exercise counselling and knowledge, and insufficient social support.^4-6^ While this work provides valuable insight into factors that limit participation, it does not provide sufficient guidance for translating exercise recommendations into sustained behavior change. The ADCES7 Self-Care Behaviors^TM^ framework emphasizes that in addition to overcoming barriers, effective diabetes self-management requires individualized goal setting, collaborative problem-solving, and tailoring strategies to personal preferences.^7^ Understanding the goals individuals hope to achieve through exercise as well as the facilitators and program characteristics that support participation may therefore provide important insight for designing more person-centered exercise counselling and interventions.

However, there is limited evidence describing the exercise goals, facilitators, and preferences that could inform exercise prescription and behavioral support specifically for females with T2D as most research has included mixed-sex cohorts without reporting sex-disaggregated findings.^8,9^ Moreover, existing research has often focused on older adults,^4^ providing limited insight into the factors influencing exercise self-management in younger/middle-aged females with T2D. PA behaviors and their determinants differ across life stages, with factors such as work, caregiving, menopause, and health status influencing priorities and opportunities to exercise.^10^ With T2D becoming increasingly common in younger adults,^11^ it is critical to understand their exercise motivations and support needs to optimize engagement and reduce long-term cardiovascular risk. Therefore, this study aimed to characterize the exercise facilitators, preferences, and goals of females with T2D and explore potential differences by age group.

## Methods

### Research Design

This is a mixed-methods study using data from a larger survey examining exercise self-management among Australian females with T2D. The full survey methodology and findings relating to exercise barriers, knowledge, and self-efficacy have been published previously.^5^ The present study reports novel analyses of exercise facilitators, preferences, and goals from the same participant cohort. Data were collected using an online survey hosted via Qualtrics (Qualtrics, Provo, Utah, USA) between November 2023 and May 2024. The survey included both quantitative (tick boxes, Likert scales) and qualitative (free-text) items to capture demographics, PA levels, exercise knowledge and perceptions, exercise self-efficacy, perceived barriers and facilitators, exercise preferences, and personal goals. The free-text question regarding personal goals was presented to all participants but was optional to complete. A mixed-methods approach was used to provide a more in-depth exploration of participants’ motivations and support needs.^12^

### Participants and Recruitment

Participants were recruited through convenience sampling via advertisements distributed on social media (Facebook, Instagram), the Diabetes Victoria website, and flyers posted around Deakin University. Interested individuals accessed the survey via a web link or QR code and self-screened for eligibility. Eligibility criteria included a self-reported diagnosis of T2D, self-identification as female, residing in Australia, and age ≥18 years. The study was approved by the Deakin University Human Ethics Advisory Group (reference: HEAG-H 116_2020). Informed consent was provided electronically prior to survey commencement.

### Survey Outcomes

#### Clinical characteristics

Clinical characteristics included self-reported age, menopausal status, height and weight (used to calculate body mass index [BMI; kg/m^2^]), T2D duration, A1C level, number of T2D medications, comorbidities, and PA status. Participants were asked whether they engaged in ≥ 150 min/wk of PA as part of their T2D management. Those responding “yes” were asked to estimate time spent in low-, moderate-, and vigorous-intensity exercise per week. Total moderate-to-vigorous PA (MVPA) per week was calculated as the sum of moderate- and vigorous-intensity PA and compared to PA guidelines for individuals with T2D.^1^

#### Exercise facilitators and preferences

Participants were asked to identify exercise facilitators from a predefined list.^9,13,14^ Participants could also specify additional factors through free-text responses. Eight questions explored exercise preferences, including frequency, intensity, duration, type, exercise setting, and level of support.

#### Exercise goals

Participants were asked the open-ended question: “What are your personal goals related to exercise and diabetes management?” Responses were collected as unrestricted free-text entries for qualitative analysis. It was not compulsory to complete this question.

### Statistical Analysis

Quantitative analyses were performed in Stata 18 (StataCorp, TX, USA). Missing data were not imputed. Normality was assessed using Shapiro-Wilk tests and visual inspection. Survey responses were analyzed using descriptive statistics to summarize participant characteristics and reported facilitators and preferences. Since not all continuous variables were normally distributed, participant characteristics are reported as both mean ± standard deviation and median (95% CIs). Categorical data are presented as frequencies (%). To explore whether specific facilitators or preferences were reported more frequently by different age groups, participants were categorized into younger (18-54 years) and older (≥55 years) groups. Group differences were analyzed using Mann-Whitney U tests for continuous variables and χ^2^ analyses for categorical variables. Fisher’s exact test was used when >20% of expected cell counts were <5. Free-text responses (goals) were categorized as binary variables (present/absent) and compared between age groups using χ^2^ analyses. A *P* value <.05 was considered statistically significant.

### Thematic Analysis

Free-text responses were analysed using thematic analysis^15^ to identify common exercise-related goals expressed by participants. Responses were imported into NVivo (QSR International, version 15) for organisation and coding. The first author familiarised themselves with the data by reading and rereading responses and then generated initial codes based on recurring words, phrases, or ideas. Codes were subsequently grouped into broader themes and subthemes that captured participants’ motivations for engaging in exercise. Representative quotations were selected to illustrate key themes.

## Results

### Participant Characteristics

A total of 119 eligible responses were included in the analysis. Participant characteristics have been reported previously.^5^ Table 1 presents the demographic and clinical characteristics relevant to the current study. Briefly, participants were between 22 and 87 years old (mean age: 59.5 ± 13.3 years), with a median T2D duration of 4.1 years (95% CI, 3-6). Most participants (71%) had an A1C <8.0% and managed their T2D through lifestyle changes (92%) and/or a single medication (53%). Most participants had at least 1 comorbidity, which was most commonly obesity (median BMI: 30.5kg/m^2^, 95% CI, 29.4-32.1). Younger females had fewer comorbidities (1 vs 3) and were predominantly premenopausal (83%). No other significant differences in characteristics or T2D management were observed between age groups (Table 1). Participants were located across Australia, with the highest representation from Victoria (40%), New South Wales (29%), and Queensland (14%).

**Table 1.** Participant Characteristics.^a^

Variable^b^	Cohort (N = 119)	Younger (n = 36)	Older (n = 83)	*P* ^c,d,e^
Age (y)	59.5 ± 13.3 63 (60-65)	42.7 ± 9.6 44 (38.6-50)	66.8 ± 6.1 67 (65-68)	**<.001**
Ethnicity, Oceanian	68 (57.1)	19 (52.8)	49 (59.0)	.526
BMI (kg/m^2^)	32.8 ± 9.8 30.5 (29.4-32.1)	34.6 ± 12.2 30.5 (28.0-35.1)	32.1 ± 8.5 30.6 (28.9-32.3)	.546
T2D duration (y)	7.3 ± 8.1 4.1 (3.0-6.1)	4.8 ± 4.7 3.3 (2.1-5.7)	8.4 ± 9.0 5 (3.0-7.9)	.103
A1C levels				.433
<5.7%	8 (7)	1 (3)	7 (8)	
5.7%-6.4%	32 (27)	9 (25)	23 (28)	
6.5%-7.9%	44 (37)	12 (33)	32 (39)	
≥8.0%	18 (15)	6 (17)	12 (15)	
Unknown	17 (14)	8 (22)	9 (11)	
Number of T2D medications, 1	63 (53)	20 (56)	43 (52)	.784
Lifestyle changes	110 (92)	33 (92)	77 (93)	.834
Number of comorbidities	3 (2-3)	1 (1-2)	3 (2-3)	**.006**
Menopausal status, Postmenopausal	88 (74)	6 (17)	82 (99)	**<.001**
Currently physically active, Yes	68 (57)	21 (58)	47 (57)	.863
MVPA (min/wk)	70 (45–106)	90 (46–138)	60 (32–116)	.440

Abbreviations: A1C, glycated hemoglobin; BMI, body mass index; MVPA, moderate-vigorous physical activity; T2D, type 2 diabetes.

a Values are presented as frequency (%) or mean ± standard deviation and median (95% confidence interval). Not reported: BMI (n = 2), comorbidities (n = 3).

b Responses with the highest proportion are reported.

c *P* values examine differences in characteristics between younger and older females.

d Bold indicates statistical significance (*P* < .05).

e For questions allowing only a single response (A1C), the reported *P* value reflects the overall chi-square comparison across groups.

### Exercise Facilitators

**Figure 1.**
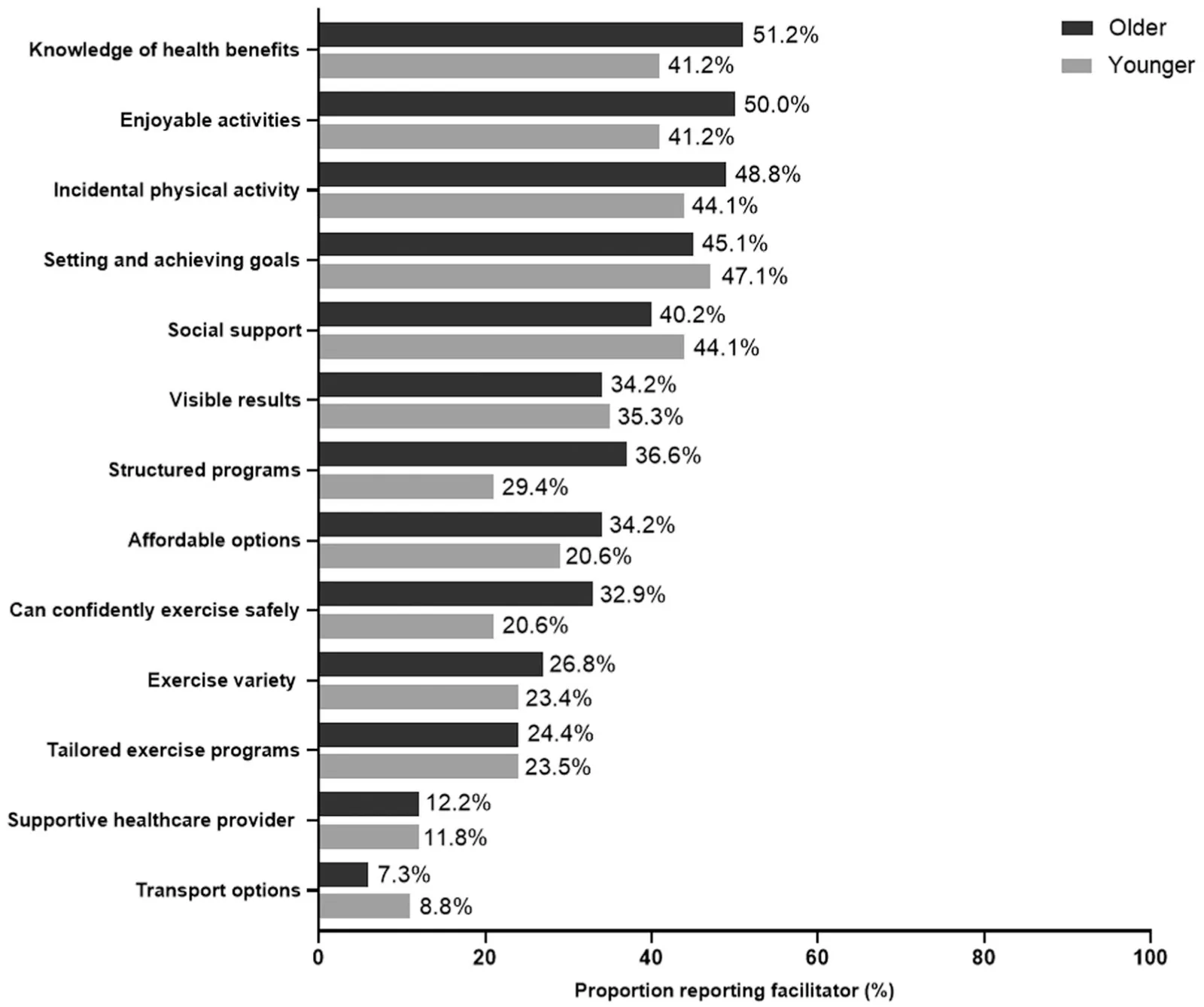
Proportions (%) of reported exercise facilitators by age group. Dark bars represent older females; light bars represent younger females.

The most frequently reported facilitators were health benefits (48%), opportunities for incidental PA (e.g., active commuting, taking the stairs; 47%), enjoyment of the activity (47%), goal setting and achievement (46%), and access to social support (41%). No significant differences in facilitators were observed between age groups (Figure 1).

### Exercise Preferences

Most participants preferred aerobic activities performed 3 to 6 days per week at moderate intensity for 30 to 45 minutes, primarily in a home setting with weekly check-ins for support. Fewer than half (46%) selected resistance training as part of their preferred routine. Preferences were generally consistent across age groups, although a near-significant trend (*P* = .05) suggested younger females were more likely to prefer vigorous-intensity exercise than those aged ≥55 years (Table 2). Few participants reported no specific exercise preferences.

**Table 2.** Exercise Preferences by Age Group in Females With Type 2 Diabetes.^a^

Exercise Preferences^b^	Response	Cohort (N = 119)	Younger (n = 36)	Older (n = 83)	*P* ^c,d^
Frequency “How many days per week would you prefer to exercise?” [3 d]	1 d	2 (2)	2 (5)	0 (0)	.166
	2 d	7 (6)	3 (8)	4 (5)	
	3 d	26 (22)	6 (16)	20 (24)	
	4 d	10 (8)	2 (5)	8 (10)	
	5 d	21 (18)	10 (27)	11 (13)	
	6 d	21 (18)	4 (11)	17 (21)	
	Daily	14 (12)	4 (11)	10 (12)	
	No preference	18 (15)	6 (16)	12 (15)	
Intensity “Which levels of intensity would you prefer to exercise at?” Select all that apply. [Moderate intensity]	Low intensity	54 (45)	18 (50)	36 (43)	.505
	Moderate intensity	86 (72)	25 (69)	61 (74)	.650
	Vigorous intensity	13 (11)	7 (19)	6 (7)	.050
	No preference	2 (2)	1 (3)	1 (1)	.540
Time “How long would you prefer to exercise for at a time?” [30-45 min]	<15 min	8 (7)	5 (14)	3 (4)	.265
	15-30 min	29 (24)	6 (17)	23 (28)	
	30-45 min	45 (38)	14 (39)	31 (37)	
	45-60 min	23 (19)	7 (19)	16 (19)	
	> 60 min	10 (8)	2 (6)	8 (10)	
	No preference	4 (3)	2 (6)	2 (2)	
Type “Which are your preferred types of exercise?” Select all that apply. [Aerobic]	Aerobic	81 (68)	25 (69)	56 (68)	.832
	Resistance	55 (46)	15 (42)	40 (21)	.512
	Combined aerobic/ resistance	29 (24)	21 (58)	8 (10)	.389
	Flexibility	54 (45)	16 (44)	38 (20)	.893
	Balance	39 (33)	8 (22)	31 (46)	.106
	No preference	16 (13)	7 (19)	9 (11)	.206
Setting “What exercise settings do you prefer?” Select all that apply. [Home]	Home	64 (53)	22 (61)	42 (51)	.291
	Outdoor activities	44 (37)	12 (33)	32 (39)	.588
	Gym	30 (25)	9 (25)	21 (25)	.972
	Group classes	48 (40)	10 (28)	38 (46)	.066
	1:1 supervision	32 (27)	8 (22)	24 (29)	.449
	No preference	9 (8)	2 (6)	7 (8)	.986
Support “What level of interaction or support do you prefer?” [Weekly check-ins]	Weekly	68 (57)	15 (42)	53 (64)	.097
	Monthly	20 (17)	10 (28)	10 (12)	
	Self-guided	16 (13)	6 (17)	10 (12)	
	No preference	15 (13)	5 (14)	10 (12)	

a Values are frequency (%).

b Responses with the highest proportion are reported in square brackets.

c *P* values examine differences between younger and older females.

d For questions allowing only a single response (frequency, time, support), the reported *P* value reflects the overall chi-square comparison across groups.

### Exercise and Type 2 Diabetes Management Goals

Of the 119 participants, 111 (93%) provided free-text responses regarding personal goals. Of the 8 participants who did not respond, most (n = 6) were from the younger cohort. Thematic analysis identified 6 themes, which are detailed below with illustrative quotations. No meaningful differences were observed between age groups.

#### Weight loss

Weight loss was the most common goal, reported by half of respondents (51%). In contrast, only a small minority (n = 4) described goals related to body composition, such as “getting toned” or “building muscle”.

Participants often linked weight loss to the potential for improved diabetes management and reduced reliance on medications:

“I want to gain fitness and stamina with the ultimate goal of weight loss. This will help me control my diabetes and possibly get off medication.” (72 years, T2D duration 8.1 years)

“To lower my diabetes and weight so I can go off medication.” (71 years, T2D duration 1.1 years)

“I would like to lose weight and maybe come off Metformin eventually.” (46 years, T2D duration 2.5 years)

Others described the goal of weight loss in relation to their overall health and well-being:

“To lose weight and improve overall fitness, which would benefit both my diabetes and overall health, both physically and mentally.” (64 years, T2D duration 28.2 years)

“My goal is being able to control my weight, so I don’t get unwell any more than I already am.” (22 years, T2D duration 3 years)

“To lose weight and feel more confident in my health and well-being.” (56 years, T2D duration 10 years)

While some participants associated weight loss with additional benefits, others described it as the primary objective:

“I want to gain fitness and stamina with the ultimate goal of weight loss.” (65 years, T2D duration 3.2 years)

“My goals are more related to weight loss and general fitness. Any flow on for my diabetes is coincidental.” (68 years, T2D duration 2.2 years)

#### Glycemic variability and diabetes management

Improved glucose management was another key objective for respondents. While some articulated specific, measurable targets, such as “stable levels under 7mmol/L” or “A1C < 6%,” many expressed more general aims around keeping their “blood sugar levels under control” and achieving “better diabetes management.” Only 3 respondents reported goals related to cardiometabolic risk factors outside of weight and glucose management (ie, blood pressure, stress, and sleep).

Respondents also linked lower glucose levels to reduced reliance on medication:

“To lose weight and sugar levels low enough to negate the need for medication.” (66 years, T2D duration 9.1 years)

“I want to be a healthy BMI, lower my sugar levels and rely less on medication.” (40 years, T2D duration 6.2 years)

Some further described the possibility of T2D remission, as reflected in goals of “reversing” or “eradicating” the condition.

#### Reducing medication

Reducing medication burden also emerged as a distinct theme, although it was typically embedded within broader goals related to weight and glycemic variability. Participants described wanting to “keep ahead of needing,” “rely less on,” or “get off” medication, often aiming to manage their T2D through lifestyle interventions alone:

“I want to get to the stage where I can control it just with diet and exercise.” (72 years, T2D duration 8.1 years, 1 T2D medication)

“My goal is to eventually stop medication and manage it by diet alone.” (73 years, T2D duration 0.2 years, 1 T2D medication)

“I want to keep from taking medications for my diabetes.” (59 years, T2D duration 3.4 years, lifestyle modifications only)

#### Increasing physical activity levels

Goals related to increasing PA levels were also commonly reported, with many participants using general expressions such as “start regular exercise,” “get moving,” or “try to be as active as possible.” Some specified quantifiable targets, such as step counts, minutes of activity per week, and exercise frequency, although exercise intensity was never mentioned.

Beyond simply increasing PA levels, participants also emphasized sustaining PA by finding personal motivation:

“I want to become more interested in exercise so I continue for a longer period of time.” (57 years, T2D duration 1.6 years)

“I want to enjoy exercise knowing that it helps with diabetes management.” (63 years, T2D duration 0.1 years)

Others emphasized the need for practical strategies to incorporate PA into their daily routines:

“To try to find more opportunities for movement (eg, walking to a social event instead of driving).” (38 years, T2D duration 1.3 years)

“To create an effective exercise regime that is easy to perform and is adaptable around my busy schedule.” (39 years, T2D duration 9 years)

#### General health and well-being

Participants often expressed broad goals focused on achieving and maintaining overall health and well-being, such as “being as healthy as possible,” “living longer,” and “being able to enjoy life.” Some also outlined more specific aspirations, such as being pain-free, reducing fatigue, or having a positive self-image.

#### Physical fitness and functional capacity

Participants also articulated broad goals aimed at enhancing their overall fitness, with expressions such as “get fit,” “stay fit,” or “continue improving fitness.” Fewer participants identified goals focused on improving specific functional capacities, such as strength, balance, and flexibility, with similarly nonspecific statements such as “gain strength,” “maintain balance,” or “achieve more flexibility in movement.”

## Discussion

This mixed-methods study examined the facilitators, preferences, and goals influencing exercise self-management among Australian females with T2D. Quantitative data indicated that participants were primarily motivated by the perceived health benefits of exercise, particularly weight loss and glucose management; however, qualitative responses revealed that these outcomes were often not viewed as independent endpoints but instead as pathways toward a broader goal of reducing the need for medication. This insight highlights an opportunity to better align diabetes self-management education and exercise counselling with person-centered priorities. Moreover, facilitators, preferences, and goals were largely consistent across age groups, suggesting that many strategies to support exercise self-management in females with T2D may be broadly applicable across adulthood.

Despite valuing exercise for its perceived health benefits, we previously reported that this cohort had limited knowledge of benefits beyond weight and glucose management.^5^ This narrow understanding was reflected in their exercise goals, with few identifying improvements in other cardiometabolic risk factors (eg, hypertension, dyslipidemia) as meaningful outcomes despite the high prevalence of these comorbidities. A similar prioritization of weight-related goals has been reported among adults attending diabetes self-management education, where weight reduction was emphasized as a strategy to improve clinical outcomes.^16^ Broadening exercise messaging to include benefits that occur even in the absence of weight loss may therefore expand the range of meaningful goals females associate with PA.

Thematic analysis provided additional context to participants’ prioritization of weight and glycemic outcomes. While these goals were often described as primary objectives, responses indicated that they were often viewed as strategies to avoid or reduce medication use. Medication burden and treatment complexity are well-documented challenges in T2D management,^17,18^ particularly among females, who experience higher rates of treatment-related adverse events.^19,20^ These findings suggest that exercise goals are often interconnected and underpinned by broader motivations, which aligns with emerging perspectives on goal setting that emphasize the need to move beyond simplistic or prescriptive frameworks (eg, SMART [specific, measurable, achievable, relevant, and time-bound] goals) and instead consider the underlying drivers, context, and meaning attached to goals.^21^ From a clinical perspective, this suggests that simply identifying an individual’s stated goals may be insufficient and that exploring why these goals are important may reveal more meaningful targets for intervention. Framing exercise as a strategy to potentially achieve medication reduction^22^ may resonate more strongly than traditional weight- and glycemia-focused messaging.

Participants preferred moderate-intensity aerobic exercise lasting 30 to 45 minutes per session, with walking as the main activity. The preferred frequency varied, but 3 days per week was the most common, which broadly amounts to the ≈120 minutes of MVPA this cohort previously identified as realistically achievable.^5^ Encouraging females with T2D to incorporate incidental activity into their daily routines (eg, walking for errands, taking the stairs, active commuting) is a practical strategy to help them reach the recommended 150 min/wk of aerobic activity. In our study, opportunities for incidental activity emerged as a key facilitator, and prior qualitative research has shown that females with T2D often conceptualize PA as part of daily routines rather than structured exercise.^13^ Therefore, strategies that emphasize incidental activity align with how females already view and undertake exercise, which could help bridge the gap between participants’ preferences, perceived capability, and guideline targets. For this approach to be effective, it should be paired with guidance on monitoring exercise intensity (eg, talk test, Borg’s rating of perceived exertion) as nearly all participants reported lacking these skills.^5^

In addition to meeting aerobic guidelines, adults with T2D are recommended to perform resistance exercise on 2 to 3 ds/wk,^1^ yet fewer than half of participants expressed an interest in this modality. This low preference may reflect perceptions of weightlifting and gym environments as more masculine and intimidating^23,24^ or a lack of knowledge and skills to engage in weightlifting confidently.^13,24^ Resistance training improves insulin sensitivity, glucose uptake, muscle mass, strength, bone mineral density, fat mass, blood pressure, and lipid profiles.^1,25^ Framing these benefits in terms of participants’ stated goals, such as glycemic variability, body composition, and functional capacity, may help enhance the perceived relevance of resistance training and support uptake. Additionally, offering resistance training through alternative formats, such as home-based programs or group classes, may improve acceptability among females who report lower interest in conventional gym-based weightlifting.

Finally, home was the most preferred exercise setting. Home-based exercise interventions have demonstrated good feasibility and adherence in adults with T2D, although improvements in clinical outcomes, such as A1C and systolic blood pressure, often depend on the provision of supervision or behavioral support.^26-28^ This is reflected in participants’ preference for weekly check-ins, suggesting a need for ongoing support; however, the specific nature of these check-ins (eg, accountability, feedback on clinical markers, encouragement, addressing safety concerns) was not captured. Given that weekly clinician-led check-ins are likely not feasible due to time and resource constraints in routine diabetes care,^29^ helping females with T2D identify sources of social support (eg, friends, family, peers) and facilitating access to group-based or community exercise options may be a practical way to maintain engagement, particularly for older females, who were more likely to report its absence as a barrier.^5^

### Strengths and Limitations

A key strength of this study is the use of a mixed-methods approach, which provided a deeper understanding of the factors supporting exercise self-management. Thematic analysis enabled the identification of nuanced patterns, such as the distinction between short-term goals (weight/glycemia) and broader motivations (reducing medication dependence). Without the qualitative data, weight loss and glycemic management may have been interpreted as participants’ primary goals rather than intermediate steps toward the broader motivation of reducing medication dependence. Another key strength is the female-only sample, which addresses an underrepresented population in exercise research. The inclusion of participants across a broad age range also allowed exploration of potential age-related differences, indicating that many strategies may be applicable across life stages.

However, several limitations should be considered. First, the survey relied on self-reported data, which may be subject to recall or social desirability bias even though responses were collected anonymously to help minimise this.^30^ Second, the use of an online survey without a defined sampling frame combined with the self-selection nature of participation may introduce sampling bias and limit the representativeness of the sample.^31^ Participants who were more engaged with or interested in exercise and diabetes self-management may have been more likely to respond. Additionally, certain groups, including ethnic minorities, were underrepresented. Third, while the inclusion of free-text responses provided valuable insight, the depth of qualitative data was limited as responses were often brief and could not be probed or clarified. Future studies using interviews or focus groups may help to better capture the complexity of participants’ motivations and experiences. Fourth, recruitment of younger females with T2D was challenging, and those who did participate were less likely to provide qualitative responses. This limited our ability to explore more nuanced age-related differences influencing PA, such as those associated with the menopausal transition. Future research should consider targeted strategies to better engage younger females with T2D.

### Implications for Practice

The findings of this study suggest several practical strategies that diabetes care and education specialists can use to better support person-centered exercise counselling for females. First, clinicians should explore not only what patients hope to achieve through exercise but also why these goals are important to them, recognizing that reducing medication reliance may be a particularly meaningful motivator. Second, exercise education should extend beyond weight and glycemic management to the broader cardiovascular and functional benefits of regular PA to help patients develop a wider range of meaningful exercise goals. Given the low preference for resistance training observed in this study, clinicians should educate females on the benefts and importance in T2D management and frame the integration of resistance training around outcomes that females value to enhance acceptability and uptake. Third, clinicians should help females identify practical opportunities to accumulate PA throughout the day, including incidental activity and home-based exercise options. Finally, facilitating access to appropriate social and community support may help sustain long-term engagement, particularly when regular clinician follow-up is not feasible.

## Conclusions

This mixed-methods study provides insight into the factors supporting exercise self-management among Australian females with T2D. While weight loss and glycemic management were the most commonly reported motivators, these were often framed as strategies to reduce reliance on medication. Exercise facilitators, preferences, and goals were largely consistent across younger and older age groups, suggesting that approaches to support exercise self-management may be broadly applicable across adulthood. These findings may help inform the development of person-centered exercise counselling and self-management support strategies for females with T2D.
